# Molecular characterization of *Tps1* and *Treh* genes in *Drosophila* and their role in body water homeostasis

**DOI:** 10.1038/srep30582

**Published:** 2016-07-29

**Authors:** Miki Yoshida, Hiroko Matsuda, Hitomi Kubo, Takashi Nishimura

**Affiliations:** 1 Laboratory for Growth Control Signaling, RIKEN Center for Developmental Biology (CDB), 2-2-3 Minatojima-Minamimachi, Chuo-ku, Kobe, Hyogo 650-0047, Japan; 2 Graduate School of Biological Science, Nara Institute of Science and Technology, 8916-5 Takayama, Ikoma, Nara 630-0101, Japan

## Abstract

In insects, trehalose serves as the main sugar component of haemolymph. Trehalose is also recognized as a mediator of desiccation survival due to its proposed ability to stabilize membranes and proteins. Although the physiological role of trehalose in insects has been documented for decades, genetic evidence to support the importance of trehalose metabolism remains incomplete. We here show on the basis of genetic and biochemical evidence that the trehalose synthesis enzyme Tps1 is solely responsible for the *de novo* synthesis of trehalose in *Drosophila*. Conversely, a lack of the gene for the trehalose hydrolyzing enzyme *Treh* causes an accumulation of trehalose that is lethal during the pupal period, as is observed with *Tps1* mutants. Lack of either *Tps1* or *Treh* results in a significant reduction in circulating glucose, suggesting that the maintenance of glucose levels requires a continuous turnover of trehalose. Furthermore, changes in trehalose levels are positively correlated with the haemolymph water volume. In addition, both *Tps1* and *Treh* mutant larvae exhibit a high lethality after desiccation stress. These results demonstrate that the regulation of trehalose metabolism is essential for normal development, body water homeostasis, and desiccation tolerance in *Drosophila*.

Organisms live in variable environments, and therefore adaptive strategies have evolved to cope with adverse environmental conditions such as starvation, low oxygen levels, and lack of water. Approximately 65 to 70% of our body weight consists of water, with one-third of the total being extracellular fluid. Energy obtained from the metabolic breakdown of nutrients such as sugar is utilized for various growth and maintenance processes. Water is not only the medium for enzymatic and chemical reactions in the body; it also moves nutrients and oxygen through the circulatory system. Therefore, maintenance of systemic water homeostasis and fluid balance is critical in most organisms, including humans, to tolerate changes in osmolarity and prevent desiccation^1^,^2^,^3^.

Trehalose is a non-reducing disaccharide that is present in a broad range of organisms including bacteria, yeast, fungi, plants, and invertebrates^4^,^5^. In insects, trehalose serves as the main sugar component of haemolymph. It is synthesized in the fat body, an organ analogous to the mammalian liver, and released into the haemolymph^6^,^7^,^8^. Trehalose has been recognized to be an important osmoprotectant and a mediator of desiccation tolerance because of its stability and inert chemical properties^9^,^10^,^11^,^12^,^13^. Many of these roles have been documented in the budding yeast *Saccharomyces cerevisiae*, where trehalose is proposed to play a protective role by functioning as a chemical chaperone, which prevents protein denaturation and aggregation and influences protein folding through trehalose-protein interactions^14^,^15^. In addition, trehalose contributes to the preservation of membrane phospholipids organization through the packing of lipid acyl chains^9^,^10^,^16^,^17^. In the sleeping chironomid *Polypedilum vanderplanki*, trehalose accumulates, making up as much as 20% of the dry body mass, and is thought to replace water in tissues to successfully achieve an anhydrobiotic state^10^,^13^. Although trehalose accumulation is not the only mechanisms involved in desiccation tolerance, it has been demonstrated to be an important factor for desiccation tolerance based on genetic evidence in diverse multicellular organisms such as the nematode *C*. *elegans*^16^, the mosquito *Anopheles gambiae*^18^, and the fruit fly *Drosophila melanogaster*^19^. The physiological role of trehalose in insects has been proposed for decades. However, genetic evidence to independently validate the importance of trehalose metabolism and the role of trehalose as a chemical chaperone remains incomplete.

Trehalose is synthesized by the trehalose synthesis enzyme Tps1 and is hydrolyzed by trehalase. Two *trehalase* genes, a soluble form called *Tre-1* and a membrane-bound form called *Tre-2* that has a transmembrane domain at the C-terminus, have been identified in many insects, including the silkworm *Bombyx mori*^20^,^21^, the bamboo borer *Omphisa fuscidentalis*^22^, the mired bug *Apolygus lucorum*^23^, and the whitefly *Bemisia tabaci*^24^. Although differences in expression pattern of the two *trehalase* genes have been reported in several insects, the functional significance of each trehalase being distributed in different locations remains unknown.

We previously reported that larvae lacking trehalose exhibit diet-dependent phenotypes relating to growth and survival in the genetically tractable organism, *D*. *melanogaster*^25^. In this manuscript, we describe the molecular characterization of the genes responsible for trehalose metabolism, *Tps1* and *Treh*. We further determined the importance of each isoform of *Treh* by generating isoform-specific mutants. These genetic mutants allow us to directly assess the physiological role of trehalose during development. We demonstrate the importance of trehalose metabolism for the maintenance of free glucose levels. In addition, defects in trehalose metabolism affect water homeostasis and desiccation tolerance.

## Results

### Tps1 is solely responsible for the *de novo* synthesis of trehalose in *Drosophila*

**Figure 1:**
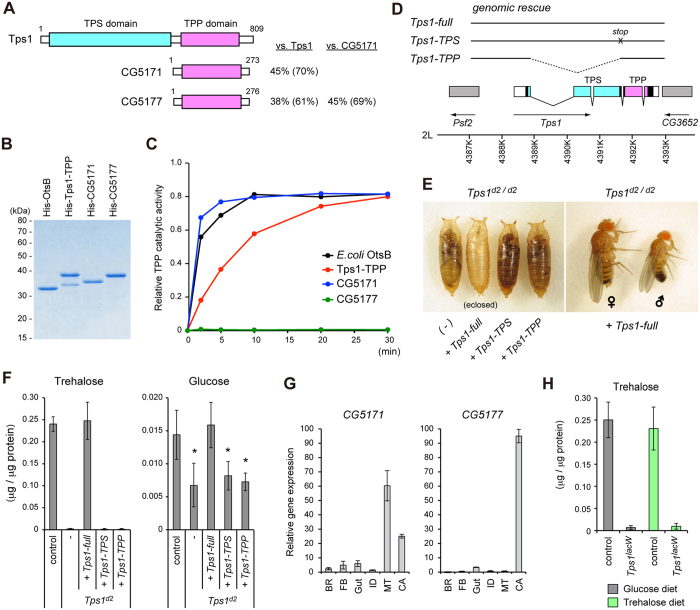
Tps1 is solely responsible for the *de novo* synthesis of trehalose in *Drosophila*. (**A**) Domain structure of Tps1 and the TPP-domain containing proteins, CG5171 and CG5177. Numbers refer to amino acids. Identities and similarities of amino acids between proteins are shown on the right. (**B**) Recombinant His-tagged proteins used in the assay were analyzed by CBB staining. (**C**) Relative TPP activity was analyzed by the incubation of a recombinant enzyme and the substrate trehalose-6-phosphate. (**D**) Schematic representation of the *Tps1* locus and molecular nature of the genomic rescue constructs. The protein-coding regions and untranslated regions are represented by filled boxes and open boxes, respectively. The transcript regions of the neighboring genes are represented by grey boxes. (**E**) Both the TPS and TPP domains are required to rescue the *Tps1* mutant phenotype from pupal lethality. (**F**) Trehalose and glucose levels were analyzed in late third instar larvae of the indicated genotypes. (**G**) The expression patterns of *CG5171* and *CG5177* were analyzed by qRT-PCR in mid third instar larvae. BR, Central nervous system; FB, Fat body; ID, Imaginal discs; MT, Malpighian tubule; CA, Carcass. Relative gene expression levels in each tissue are shown. Total values are set to 100. (**H**) The food containing trehalose instead of glucose did not rescue the trehalose levels in the *Tps1* mutant wandering larvae. All the values are means and SD (n = 6 [**F**,**H**] or n = 3 [**G**]). Statistical significance is determined by two-tailed Student’s *t*-test (*P* < 0.05).

The gene for the trehalose synthesis enzyme, *Tps1*, is exclusively expressed in the fat body^25^. We have previously shown that the knockdown of *Tps1* in the fat body fully recapitulates the ubiquitous knockdown phenotype^25^. Tps1 has two functionally distinct catalytic domains^4^,^5^. The N-terminal TPS (trehalose-6-phosphate (T6P) synthase) domain catalyzes the production of T6P using glucose-6-phosphate and UDP-glucose. The C-terminal TPP (T6P phosphatase) domain then dephosphorylates T6P to generate trehalose. We failed to detect genes other than *Tps1* in the *Drosophila* genome that possess a TPS domain, whereas there are two uncharacterized genes containing only a TPP domain: *CG5171* and *CG5177* (Fig. 1A). Similarly, there are multiple genes in *C*. *elegans*, plants, and bacteria that contain only a TPP domain in addition to Tps1^5^,^26^,^27^,^28^. It has been shown that the TPP domains of Tps1 in some plant species have lost their catalytic activity^29^. These observations raise a question about the necessity of the C-terminal TPP domain in the *Tps1* gene product in *Drosophila*.

To address this issue, we directly assessed the phosphatase activity of the TPP domain of *Drosophila* Tps1 *in vitro*. A bacterially expressed Tps1-TPP was purified, and its enzymatic activity was compared with those of CG5171, CG5177, and an *E*. *coli* gene product, OtsB, which possesses the TPP domain (Fig. 1B). We found that recombinant Tps1-TPP and CG5171, but not CG5177, dephosphorylated T6P under our experimental conditions (Fig. 1C). Time-course analyses revealed that Tps1-TPP showed a lower activity than CG5171 and OtsB *in vitro*. The enzymatic activity of Tps1 may be post-translationally regulated via mechanisms such as phosphorylation and methylation, as has been demonstrated in yeast and bacteria^4^,^30^.

If CG5171 can substitute for the function of TPP in Tps1, the TPS domain of Tps1 may be sufficient to rescue the mutant phenotype in *Tps1* null mutants. To further confirm the necessity of the TPS and TPP domains of Tps1 *in vivo*, we generated genomic-rescue constructs with a full-length *Tps1* gene, a *Tps1* gene with a stop codon before the TPP domain, and a *Tps1* gene lacking the TPS domain (Fig. 1D). One-copy of the full-length *Tps1* genomic construct completely rescued the lethality of the *Tps1* mutants, whereas neither the TPS domain nor the TPP domain of *Tps1* rescued the mutant phenotype (Fig. 1E). Consistently, the reductions of the trehalose and glucose levels in the *Tps1* mutants were not rescued by the TPS domain nor the TPP domain of *Tps1* (Fig. 1F). These results indicate that the TPS and TPP domains in *Tps1* are both required for trehalose synthesis *in vivo*. We observed that *CG5171* was mainly expressed in the Malpighian tubules and the components of the carcass, which included body wall muscles, but not in the fat body (Fig. 1G). The difference in the expressing tissues likely explains the *in vivo* requirement of the TPP domain of Tps1. Furthermore, feeding trehalose to the flies failed to restore trehalose levels or rescue pupal lethality in the *Tps1* mutants (Fig. 1H), suggesting that trehalose is not directly supplied from the food through the gut in flies. Alternatively, the absorption rate of trehalose through the gut may be low, and therefore dietary trehalose is not enough to restore the normal levels of circulating trehalose. Taken together, these results indicate that *Tps1* is solely responsible for the *de novo* synthesis of trehalose in *Drosophila*.

### *Treh* mutations are lethal in the pupal period

**Figure 2:**
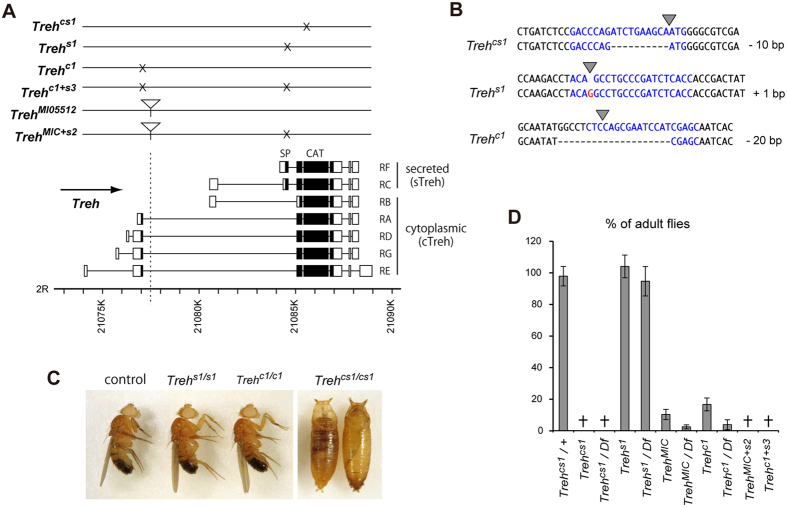
Generation and characterization of the *Treh* mutants in *Drosophila*. (**A**) Schematic representation of the *Treh* locus and the molecular nature of the mutants. Protein-coding regions and untranslated regions are represented by filled boxes and open boxes, respectively. The *P*-element insertion sites are marked with an inverted triangle. Each transcript is based on information from FlyBase. (**B**) Sequences of sgRNA target sites and the deletion/insertion of *Treh* mutants. The 20 bp target sequence corresponding to each target site is indicated in blue and the cleavage sites of Cas9 are shown as triangles. (**C**) Trehcs1 homozygous mutants were lethal at the pupal period. Trehs1 mutant adult and Trehc1 escaping adult are shown. (**D**) Lethality of the *Treh* mutants. Percentages of the indicated mutant adults were determined based on the ratio of heterozygous mutants in each vial. All the values are means and SD (n > 6 [**D**]).

We next examined the functional significance of the trehalose hydrolyzing enzyme. In the *Drosophila* genome, there are two genes that possess a catalytic domain for trehalose hydrolysis: *Treh* and *CG6262*. Because *CG6262* is dominantly expressed in adult testis (Flybase), *Treh* is thought to be the main enzyme that catalyzes trehalose during development. To determine whether *Treh* plays an essential role in trehalose metabolism and development, we generated *Treh* mutants using CRISPR/Cas9 technology. We isolated several *Treh* mutant strains with frameshift deletions/insertions at the section of the catalytic domain nearest to the N-terminal using three distinct single-guide RNAs (sgRNAs). Homozygous mutants of the isolated frameshift mutants generated from each sgRNA construct survived the larval period but displayed complete lethality during pupal period. Hereafter, we used one of the deletion alleles of *Treh*, named *Treh*^*cs1*^ (Fig. 2A). *Treh*^*cs1*^ lacks 10 bp, resulting in an unusual transcript with a premature stop codon and a lack of the entire catalytic domain (Fig. 2B). Transheterozygotes of *Treh*^*cs1*^ with a deficiency allele lacking the *Treh* locus survived the larval period and died at the pupal stage (Fig. 2C,D), suggesting that the *Treh*^*cs1*^ allele is functionally null. Taken together, these results indicate that *Treh* is essential for pupal development, and the *Treh* mutants display lethality at a stage identical to that of the *Tps1* mutants that we have previously reported on ref. 25.

### Cytoplasmic Treh rather than secreted Treh is critical for normal development

Treh in *Drosophila* is thought to be produced in two different forms via variations in alternative splicing (Flybase): a putative secreted form (sTreh), with a signal peptide at the N-terminus, and a cytoplasmic form (cTreh), without a signal peptide (Fig. 2A). Both sTreh and cTreh share the same catalytic domain for trehalose hydrolysis. Both *sTreh* and *cTreh* transcripts are initiated at several different transcriptional start sites, and therefore various transcripts (5 corresponding to cTreh and 2 corresponding to sTreh) are described in the Flybase. To examine the functional importance of cTreh and sTreh, we next examined the mutant phenotype of an available *Minos*-transposon in the *Treh* locus (*MI*05112, hereafter named *Treh*^*MIC*^). The transposon is inserted in the first intron of the coding region of *cTreh* (Fig. 2A). The *Treh*^*MIC*^ mutants displayed lethality in the pharate adult stage. However, adult escapers were observed under regular rearing conditions (Fig. 2D). The number of adult escapers was significantly reduced in *Treh*^*MIC*^ transheterozygotes with the deficiency allele, suggesting that *Treh*^*MIC*^ is a hypomorphic allele.

Because *Treh*^*MIC*^ likely affects the major *cTreh* transcripts, including RA, RD, RG, and RE, but not RB, and the *sTreh* transcripts, *Treh*^*MIC*^ is likely to be a strong allele of *cTreh*. It is possible that *cTreh* functions redundantly with *sTreh*. To address this possibility, we used the CRISPR/Cas9 technique to generate *sTreh*-specific mutants by introducing small deletions at the region within the signal peptide sequence (Fig. 2A). All isolated mutants with frameshift deletions/insertions displayed homozygous viability, were fertile, and had no visible lethality during development. Hereafter, we used one of the deletion allele of *sTreh*, named *Treh*^*s1*^, which has a frameshift insertion of 1 bp (Fig. 2B,C). *Treh*^*s1*^ transheterozygotes with the deficiency allele also exhibited no lethality (Fig. 2D), indicating that *sTreh* is dispensable for successful development. Then, we introduced a similar frameshift deletion/insertion at the signal peptide sequence in the *Treh*^*MIC*^ allele using the identical *sgRNA* construct (Fig. 2A). Homozygous *Treh*^*MIC*^ mutants having a small deletion within the *sTreh* transcripts (hereafter named *Treh*^*MIC*+*s2*^) displayed complete lethality during pupal period (Fig. 2D). To further validate these results, we also introduced a small deletion at the first exon of the major *cTreh* transcripts (hereafter named *Treh*^*c1*^). Homozygous *Treh*^*c1*^ mutants displayed pupal lethality, with some adult escapers and with results similar to those observed with the *Treh*^*MIC*^ allele. The introduction of a small deletion at the signal peptide sequence on the *Treh*^*c1*^ allele (hereafter named *Treh*^*c1*+*s3*^) exhibited complete pupal lethality (Fig. 2A,D). These results indicate that *cTreh* and *sTreh* function redundantly, although the function of *cTreh*, rather than *sTreh*, is dominant within the *Treh* gene locus.

### *Treh* is required for the maintenance of glucose levels and starvation tolerance

**Figure 3:**
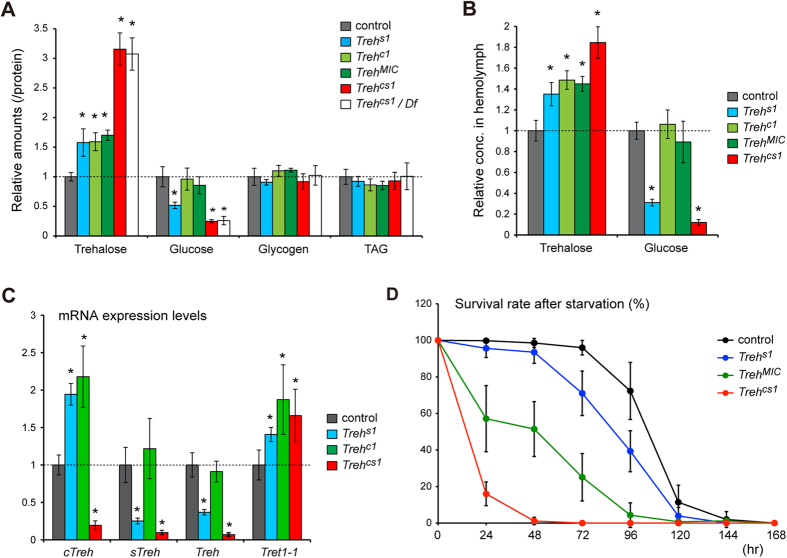
*Treh* mutations affect trehalose and glucose levels in *Drosophila*. (**A**) *Treh* mutations increase trehalose level in late third instar larvae. Each value was normalized by protein levels and further normalized according to the level in the control larvae. (**B**) Trehalose and glucose concentrations in the haemolymph were analyzed in late third instar larvae of the indicated genotypes. (**C**) *Treh* transcript levels were analyzed by qRT-PCR at the mid third instar stage of the indicated genotypes. (**D**) *Treh* mutants exhibited lethality under starvation conditions. Early third instar larvae were transferred to a vial containing 0.8% agar in PBS, and the number of surviving larvae were counted at the indicated time points. Statistical significance is determined by two-tailed Student’s *t*-test (*P* < 0.01). All the values are means and SD (n > 4 [**A**,**B**,**D**] or n = 3 [**C**]).

To further characterize the *Treh* mutants, we next analyzed trehalose levels. *Treh*^*cs1*^ mutant larvae exhibited significant increase in trehalose at the late third instar larval stage (Fig. 3A). Furthermore, trehalose concentrations in the circulating haemolymph of the *Treh*^*cs1*^ mutants were also up-regulated, although the degree of increase was less drastic when compared with the increases in the whole larvae (Fig. 3B). Consistent with the observed lethality of the mutations, *Treh*^*MIC*^ and *Treh*^*c1*^ mutant larvae displayed a partial increase in trehalose levels when compared with the increases in the *Treh*^*cs1*^ mutants. We next examined glycogen and triacylglycerol (TAG) levels to understand the metabolic consequences of defective trehalose catabolism in flies; however, the levels were not significantly altered in the *Treh* mutants (Fig. 3A). Conversely, glucose levels in the *Treh*^*cs1*^ mutants were significantly lower. *Treh*^*s1*^ mutants had increased trehalose levels, similar to *cTreh* mutants, whereas glucose levels were reduced only in s*Treh* mutants and not in *cTreh* mutants. Circulating glucose in the haemolymph was also decreased in *Treh*^*cs1*^ and *Treh*^*s1*^ mutants (Fig. 3B). The reduction of glucose levels in *Treh* mutants is reminiscent of the *Tps1* mutant phenotype (Fig. 1F)^25^, suggesting that the maintenance of glucose levels requires a continuous turnover of trehalose, including its synthesis and breakdown. Specifically, *sTreh* rather than *cTreh* plays an important role in regulating the levels of free glucose. Nevertheless, given that *sTreh* mutants displayed no lethality, severe dysregulation of glucose levels in the haemolymph does not appear to be directly related to pupal lethality in *Treh*^*cs1*^ mutants.

Our results demonstrate that *Treh*^*cs1*^ mutants exhibit a significant increase in trehalose levels in the whole larvae, which is most likely not additive between *cTreh* and *sTreh* mutants. These observations raise a possibility of a compensatory mechanism between *cTreh* and *sTreh*. We observed that *Treh* transcript levels were significantly reduced in *Treh*^*cs1*^ mutants based on a qRT-PCR analysis with primer sets that amplified the regions outside the small deletions (Fig. 3C). More specifically, *sTreh* transcripts were significantly down-regulated in *Treh*^*s1*^ mutants, suggesting positive feedback regulation in response to its own enzyme activity. On the other hand, the expression of *cTreh* was up-regulated in both *Treh*^*s1*^ and *Treh*^*c1*^ mutants, suggesting negative feedback regulation at the level of transcription and/or depending on the stability of mRNA to compensate for a defect in trehalose hydrolysis. Expression of the trehalose transporter *Tret1-1* was also up-regulated in *Treh* mutants, suggesting that feedback regulation co-operates not only in *Treh* but also at the level of trehalose transport. The compensatory up-regulation of *cTreh* and *Tret1-1* may in part account for the viability of the *Treh*^*s1*^ mutants.

To understand the role of *Treh* under conditions of dietary stress, we analyzed the starvation tolerance of *Treh* mutants. The control larvae survived for 4 days on a water-only diet, whereas almost all *Treh*^*cs1*^ mutant larvae died 2 days after the initiation of starvation conditions (Fig. 3D). *Treh*^*MIC*^ mutants exhibited a weaker but still apparent lethality under starvation conditions, whereas *Treh*^*s1*^ mutants showed only a slight reduction in survival rate when compared with the control larvae. Furthermore, *Treh*^*cs1*^ mutants failed to grow to pupae under feeding conditions that lacked dietary glucose (data not shown), which is similar to the results for *Tps1* mutants^25^. These results suggest that *Treh* mutants are sensitive to the levels of dietary glucose for their survival and larval growth. Because the reliance on dietary glucose for survival and growth in *Treh* mutant larvae is consistent with the results observed in *Tps1* mutants^25^, the observed phenotypes related to starvation and low-dietary glucose levels are due to defects in trehalose metabolism rather than to an over-accumulation of trehalose.

### *Treh* is responsible for trehalose catabolism *in vivo* and *in vitro*

**Figure 4:**
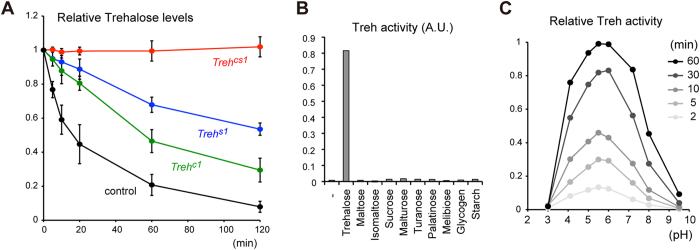
*Treh* is responsible for trehalose catabolism *in vivo* and *in vitro*. (**A**) Trehalase activity was measured in larval homogenates. Relative levels of trehalose are shown for each mutant. (**B**) Recombinant His-tagged *Drosophila* Treh specifically hydrolyzed trehalose *in vitro*. (**C**) Trehalase activity was observed in a broad range of pH conditions. Relative Treh activity as determined by the hydrolysis of trehalose is shown over time. All the values are means and SD (n = 3 [**A**]).

We next measured the trehalase activity in larval homogenates to directly test the role of *Treh* in trehalose hydrolysis. Wild-type homogenates exhibited an enzyme activity with the hydrolysis of trehalose occurring in a time-dependent manner (Fig. 4A). On the other hand, *Treh*^*cs1*^ mutant larvae had no detectable trehalose hydrolysis activity, indicating that Treh is solely responsible for the hydrolysis of trehalose at this developmental stage. Consistent with the functional redundancy between sTreh and cTreh, *sTreh* and *cTreh* mutants showed a partial trehalose hydrolysis activity under these conditions. To further characterize the *Treh* gene product, we produced recombinant His-tagged cTreh proteins in *E*. *coli*. The recombinant cTreh exhibited a specific breakdown of trehalose to produce glucose *in vitro* (Fig. 4B). The activity of His-cTreh was observed *in vitro* between pH 4.0 and pH 8.0, with a maximum activity at pH 5.5 ± 0.5 (Fig. 4C). Because the catalytic domain of Treh is shared between sTreh and cTreh, these results indicate that *Treh* is responsible for trehalose catabolism *in vivo* and *in vitro*.

### Changes in trehalose levels influence the water levels in the haemolymph

**Figure 5:**
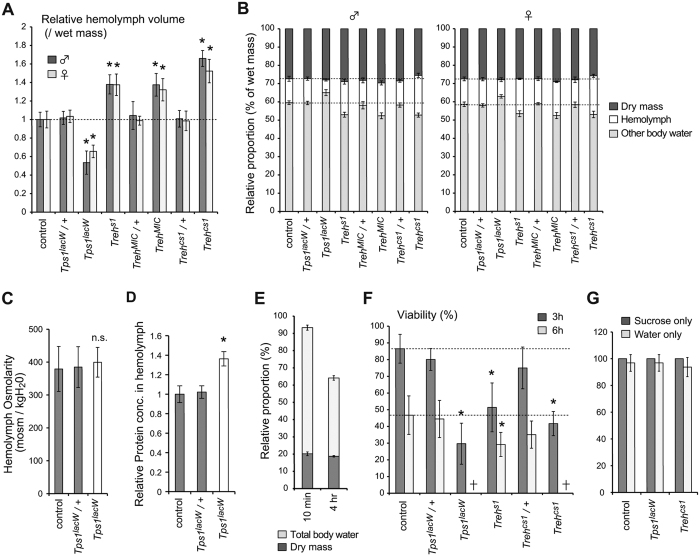
Trehalose metabolism affects haemolymph water volume and desiccation tolerance. (**A**) *Tps1* and *Treh* mutations affect the volume of haemolymph at the wandering stage. The weights of haemolymph in the indicated genotypes were measured and normalized according to wet weight. Heterozygous mutants are included to validate the accuracy of the measurements. (**B**) Relative proportions of haemolymph volume, dry weight, and the predicted water content in tissues are shown. (**C**) *Tps1* mutants exhibited no change in haemolymph osmolarity. (**D**) Protein concentrations in the haemolymph were analyzed in late third instar larvae of the indicated genotypes. (**E**) Desiccation reduced water contents in early third instar larvae. Relative proportions of water content and dry weight were normalized to the values of wet weight before desiccation. (**F**) Survival rates after desiccation and rehydration. Early third instar larvae were used to test 3 hr or 6 hr of desiccation followed by rehydration. (**G**) Survival rates under sucrose or water-only dietary conditions. Early third instar larvae were kept under each condition for 12 hours. Statistical significance is determined by two-tailed Student’s *t*-test (*P* < 0.01). All the values are means and SD (n > 6 [**A**–**D**] or n > 3 [**E**–**G**]).

During the course of experiments, we noticed that the volume of haemolymph was considerably higher in *Treh* mutants than in the control larvae. In contrast, *Tps1* mutants appeared to retain less haemolymph based on the difficulty in collecting larval haemolymph from these mutants. These observations regarding haemolymph volume were confirmed by quantifying the weight of the haemolymph (Fig. 5A). The increase in haemolymph water volume explains why the changes in trehalose concentrations in the haemolymph of *Treh*^*cs1*^ mutants were less drastic than the increase in trehalose detected in the whole larvae. Body water is the primary determinant of the osmolality of the extracellular fluid, and trehalose is known to be an organic osmoprotectant in several organisms^5^,^9^,^10^,^11^,^12^. Therefore, alterations of trehalose levels likely affect osmolality, thereby changing the water content of the haemolymph. To further examine the mutant phenotype, we next examined whether defects in trehalose metabolism altered water content at the whole-organism level. *Tps1* mutants did not exhibit a significant decrease in the percentage of water in whole larvae (Fig. 5B). These results suggest that *Tps1* mutants retain more intracellular fluid at the expense of the reduction of water in the haemolymph. Because of the size difference between males and females at the wandering stage, we analyzed the haemolymph volume and dry mass separately in males and females. We found that the relative proportion of dry mass and haemolymph volume per wet mass was relatively comparable between sexes. Our results suggest that changes in the trehalose levels affect body water homeostasis equally in males and females. We also analyzed the trehalose and glucose levels in males and females and found no significant differences between sexes (data not shown).

Importantly, we failed to detect a decrease in osmotic pressure in the haemolymph of *Tps1* mutants (Fig. 5C), even though these mutants retained less haemolymph than the controls. *Tps1* mutants showed a higher concentration of total protein in their haemolymph (Fig. 5D). These results support the possibility that the reduction of haemolymph water volume in *Tps1* mutants is the result of alterations in osmotic pressure. Conversely, *Treh* mutants showed a significant decrease in tissue water content instead of an increase in haemolymph volume (Fig. 5B). Furthermore, *Treh*^*cs1*^ mutants displayed a slight reduction in solute levels relative to their water content, suggesting a relative increase in total body water. Taken together, these results indicate that trehalose levels are positively correlated with extracellular fluid volume due to alterations in osmotic pressure.

### Importance of trehalose metabolism in desiccation tolerance

A strong relationship has been observed between haemolymph volume, carbohydrate levels, and survival during desiccation in a *D*. *melanogaster* population selected for desiccation resistance^31^. Because the genetic manipulation of trehalose levels affects water levels in haemolymph, we next examined the sensitivity of *Tps1* and *Treh* mutants to desiccation stress. During the desiccation process, larvae gradually stop moving and reduce their body size. An approximately 35% reduction in water contents in whole larvae was observed 4 hours after desiccation under our experimental conditions (Fig. 5E). In contrast, dry weight was reduced by 7% during that period. More than 80% of the wild type early 3^rd^ instar larvae recovered with rehydration after 3 hours of desiccation (Fig. 5F). Approximately 40% recovered after 5 hours, and 15% recovered after 7 hours of desiccation. Under these conditions, *Tps1* mutants exhibited a higher lethality than the heterozygous mutants and wild-type larvae. Similarly, *Treh*^*cs1*^ mutants exhibited a higher lethality rate, similar to that in *Tps1* mutants, after rehydration. We showed that both *Tps1* and *Treh* mutants are sensitive under starvation conditions (Fig. 3D)^25^. To confirm whether the observed lethality is due to the desiccation stress rather than starvation stress, we analyzed the starvation phenotype for a short period. More than 90% of mutant larvae survived under water-only or sucrose-only dietary conditions for 12 hours (Fig. 5G), indicating the specific phenotype relates to desiccation but not starvation under these conditions.

**Figure 6:**
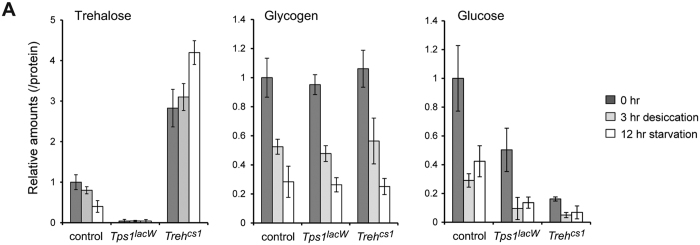
Changes in sugar levels following desiccation and starvation stress. (**A**) The amounts of trehalose, glycogen, and glucose were analyzed in early third-instar larvae following desiccation and starvation stress. Each value was normalized by protein levels and further normalized according to the level in the control larvae. All the values are means and SD (n > 3).

To further understand the importance of trehalose metabolism under stress conditions, we analyzed the sugar levels in *Tps1* and *Treh* mutants following desiccation and starvation stress. We found that *Treh* mutants further increased the amount of trehalose after 12 hours of starvation, but not after 3 hours of desiccation (Fig. 6), indicating the active production of trehalose by Tps1 following starvation stress. Glycogen and glucose were significantly reduced after 3 hours of desiccation, whereas trehalose was slightly reduced. In addition, free glucose was more reduced after 3 hours of desiccation than it was after 12 hours of starvation. *Tps1* and *Treh* mutants retained less glucose than did the control. Both desiccation and starvation further reduced the glucose levels in *Tps1* and *Treh* mutants. Conversely, the changes in the glycogen levels in *Tps1* and *Treh* mutants were almost comparable to those observed in the control. These results raise the possibility that *Tps1* and *Treh* mutants are sensitive following desiccation stress due to a reduction in the free glucose level in addition to the defects in body water homeostasis. Taken together, these results suggest that the physiological role of the trehalose pathway is closely related to metabolic regulation and that desiccation tolerance is not attributable simply to increased concentrations of trehalose.

## Discussion

Trehalose is synthesized in a two-step enzymatic reaction. In this study, we showed that both the TPS and the TPP domain in Tps1 are required for the *de novo* synthesis of trehalose in *Drosophila*. Although the function of the TPP domain proteins CG5171 and CG5177 remains unknown, the TPP domain of Tps1 is functional and solely involved in trehalose synthesis in the fat body. Interestingly, T6P is not just a precursor of trehalose in the biosynthetic pathway, it also controls the flux of glycolysis as a signaling molecule in yeast and plants^32^,^33^. In addition, T6P acts as an endogenous inhibitor of SNF1-related protein kinase in plants and controls metabolism and growth in response to starvation^34^. Glucose is directly or indirectly sensed in several ways to control cellular energy metabolism^35^,^36^,^37^. Likewise, it is possible that a metabolite such as T6P and/or trehalose functions as a signaling molecule in addition to serving as an energy source. T6P can be detected in adult haemolymph at a relatively low level in *Drosophila*^38^. It will be interesting to investigate the function of T6P in response to environmental changes in *Drosophila*.

We found that cTreh, and not sTreh, is critical for normal development, although to some extent, sTreh and cTreh function redundantly. The trehalose transporters in *Drosophila*, *Tret1-1* and *Tret1-2*, are expressed in both the fat body and in several peripheral tissues^25^,^39^. Therefore, it is likely that cTreh hydrolyzes trehalose within cells. In contrast, sTreh plays an important role for the maintenance of glucose levels in the haemolymph and in body as a whole. However, defects in cTreh have little effect on circulating glucose levels. These observations suggest that the hydrolysis of trehalose in circulating haemolymph directly influences the levels of free glucose. Furthermore, the co-existence of the enzyme and its substrate in the haemolymph has been suggested as a possible regulatory mechanism of trehalase, along with inhibitory proteins^22^,^40^,^41^. Therefore, it is thought that sTreh activity is post-translationally regulated by unknown mechanisms. Our results also suggest that the expression of *sTreh* is positively regulated by its own activity, while cTreh expression is regulated in a compensatory manner. Nevertheless, the reduction of free glucose levels in *Treh* mutants does not directly correlate with the observed lethality of the mutation. It has been reported that soluble and membrane-bound trehalase exhibit dynamic changes in expression patterns during metamorphosis^20^,^22^,^42^. Consistently with these findings, the amount of trehalose is more rapidly decreased than that of glycogen and TAG during metamorphosis^25^. Further analysis will be required to understand a precise role of trehalose during the pupal period.

Depletion of body water leads to a deleterious effect on organisms. Our results demonstrate that the haemolymph sugar trehalose significantly influences the volume of haemolymph and thereby is important for regulating the intracellular fluid. Although trehalose is thought to play a protective role under desiccating conditions, our results suggest that trehalose metabolism rather than or in addition to the presence of trehalose itself is critical for desiccation tolerance in *Drosophila* larvae. Consistent with this hypothesis, the importance of trehalose metabolism for desiccation tolerance has been documented in yeasts^43^. It is also important to consider direct versus indirect effects of trehalose on biological systems^44^.

The excretory system, composed of the kidneys in mammals and of the Malpighian tubules and the hindgut in insects, functions to maintain systemic water homeostasis^45^,^46^. Ion and water balance in insects is regulated by the balance between excretion by the Malpighian tubules and absorption by the hindgut and rectum^47^. The regulation of cellular ion and water homeostasis in the Malpighian tubules has been proposed to be a key physiological mechanism underlying stress tolerance, such as resistance to desiccation^47^,^48^,^49^. In the brown planthopper, Malpighian tubules function in the reabsorption of trehalose by a proton-dependent trehalose transporter^50^. The Malpighian tubules in several insect species exhibit high expression levels of *trehalase*^23^,^42^. Consistent with this, we previously reported that *cTreh* is highly expressed in the Malpighian tubules during the larval period^25^. However, this expression was almost completely down-regulated at the white pupal stage (data not shown). In addition to the reabsorption of trehalose, the high level expression of *cTreh* in the Malpighian tubules suggests the importance of trehalose as an energy source for maintaining organ function in the tubules, and it may indirectly contribute to water homeostasis and osmoregulation. Of note, mammals lack the gene *Tps1* but retain *trehalase*. *Trehalase* is highly expressed in the kidney brush border membranes, although the function and physiological relevance of this expression are still not clear^5^. In this sense, it will be interesting to investigate the local requirements of *Treh* in the Malpighian tubules in relation to body water homeostasis.

## Materials and Methods

### *Drosophila* strains

*Drosophila melanogaster* flies were reared on a standard agar-yeast-cornmeal medium at 25 °C. Details of the composition of the food have been described previously^25^. For trehalose feeding experiments, 10 g glucose in the food was substituted for 10 g trehalose (Hayashibara Co.) per 100 ml. All experiments were conducted under non-crowded conditions. No yeast paste was added to the fly tubes for any of the experiments. The strain *w*^*1118*^ was used as a control. *Tps1*^*lacW*^ and *Tps1*^*d2*^ have been described previously^25^. *Mi{MIC}Treh*^*MI05512*^ and *Df*(*2R*)*Exel6072* (a deficiency strain with a deleted genomic region including the *Treh* locus) were obtained from the Bloomington *Drosophila* Stock Center. *y*^*2*^ *cho*^2^ *v*^*1*^*; attP40{nos-Cas9}/CyO* and *y*^*2*^ *cho*^*2*^ *v*^*1*^, *P{nos-Cas9*, *y*^+^, *v*^+^*}/FM7c*, *KrGal4*, *UAS-GFP* were obtained from the National Institute of Genetics *Drosophila* Stock Center. *Mi{MIC}Treh*^*MI05512*^ and *Df*(*2R*)*Exel6072* were back-crossed three times with the *y*^−^ *w*^−^ and *w*^−^ strain, respectively, and were used for the experiments. The back-crossed *Mi{MIC}Treh*^*MI05512*^ was renamed as *Treh*^*MIC*^.

### Generation of the *Treh* mutants

Generation of the *Treh* allele was carried out using the CRISPR/Cas9 system with the pBFv-U6.2 vector^51^. Sense and antisense oligonucleotides corresponding to sgRNA target sequences were annealed and inserted into *BbsI*-digested pBFv-U6.2 vectors. *Treh* sgRNA vectors were injected into embryos carrying attP2 and *nos-phiC31* and the transgenic strains were generated (BestGene, Inc). The *nos-Cas9*-based gene targeting was carried out as previously described^51^. Independent isogenized strains for each sgRNA construct were established. Indel mutations were analyzed via genome DNA extraction and PCR amplification of the DNA fragment including the target site, followed by sequence analysis. We isolated several frameshift mutations for *Treh* by using three different sgRNA that target the catalytic domain of Treh. All of the isolated *Treh* mutants are pupal-lethal. Likewise, we isolated several frameshift mutations for *sTreh* by using three different sgRNAs that target the region within the signal peptide. All of the isolated *sTreh* mutants are viable and fertile. We isolated three frameshift mutations for *cTreh* by using one sgRNA, and all of them are pupa-lethal with some escapers. We chose 1 strain for each target site for further analyses and renamed them *Treh*^*cs1*^, *Treh*^*s1*^, and *Treh*^*c1*^. *Treh*^*MIC*^ and *Treh*^*c1*^ mutants were further crossed with flies carrying *sTreh* sgRNA and *nos-Cas9* to introduce indel mutations in *sTreh*.

### qRT-PCR analysis

qRT-PCR analyses was done as described previously^52^. The primers used to detect *sTreh*, *Tret1-1*, and *rp49* levels are described previously^25^. The primers used are as follows: *Treh* (*common*) sense primer: 5′-TGGGCACCGATGCAGTACATCCTG-3′, *Treh* (*common*) antisense primer: 5′-CCGAACTCATCGGCGTTGTACTTC-3′, *cTreh* sense primer: 5′-ATACGGCAGTGATCAAATCGAGTG-3′, *cTreh* antisense primer: 5′-TGCGACAAAGACTGTTGTTTCCTG-3′, *CG5171* sense primer: 5′-CTTCGGAGATCTGCACAAAGTTCG-3′, *CG5171* antisense primer: 5′-AAGCACTCTCATGGCATCTTCATCG-3′, *CG5177* sense primer: 5′-CAAGTTGAAGGCCAAGCTGATTGC-3′, *CG5177* antisense primer: 5′-CATAGACGATCTTCAGGTTCTTGG-3′.

### Plasmid construction

The cDNAs encoding *Tps1*, *CG5171*, *CG5177*, and *Treh* were cloned by RT-PCR using sequenced strains obtained from the Bloomington Stock Center. The DNA fragments of the *Tps1* genomic region were cloned by genomic PCR using the same strains. The cDNA encoding *OtsB* was cloned by genomic PCR using the *E*. *coli* strain *DH5a*. All PCR fragments were validated by DNA sequencing. *Tps1* genomic fragments were subcloned into pCaSpeR4 vectors. Transformants were obtained using a standard injection method (BestGene, Inc). We analyzed 7 independent transformants inserted into the third chromosome for each rescue construct and obtained the same results. For bacterial expression, pET28a vectors containing an N-terminal His-tag (Novagen) were used.

### Protein production and *in vitro* assays

His-tagged proteins were produced in *BL21*(*DE3*) cells, purified on Ni-NTA agarose (QIAGEN), and dialyzed in PBS. The reaction for TPP was carried out in a 30 μl assay mixture containing 20 mM Na_2_HPO_4_ (pH 7.4), 2 mM NaH_2_PO_4_, 1.8 mM KH_2_PO_4_, 132 mM NaCl, 2.7 mM KCl, 5 mM MgCl_2_, 0.2 mg/ml BSA, 0.5 mM T6P (SIGMA), and 0.02 nmol of recombinant enzyme. Reactions were started with the enzyme at 30 °C and stopped after appropriate times by placing the tubes at 100 °C for 5 min. After cooling to room temperature, the amounts of trehalose were determined using a glucose assay kit (SIGMA) after the treatment with trehalase (SIGMA). Net activities were calculated by subtracting the values without treatment with trehalase.

The reaction for Treh was carried out in a 15 μl assay mixture containing 5 μg sugar and 0.12 pmol of His-cTreh in PBS. After an incubation at 30 °C for 2 hours, the amounts of glucose were determined using a glucose assay kit (SIGMA). For the measurement of Treh activity, the reaction was carried out in a 20 μl assay mixture containing 5 μg trehalose and 0.01 pmol His-cTreh under various buffer conditions as follows; pH 3.0 (20 mM ammonium formate buffer), pH 4.1, pH 5.0, and pH 5.5 (20 mM sodium acetate buffer), pH 6.0 and pH 7.2 (20 mM phosphate buffer), pH 8.0 and pH 9.5 (20 mM ammonium bicarbonate buffer). After the incubation at 30 °C for the indicated period, the reaction was stopped by heat inactivation at 90 °C for 5 min. The amounts of glucose were determined using a glucose assay kit (SIGMA). Because of the excess amount of the glucose assay solution relative to the volume of reaction mixture, the detection of glucose was not affected by the different pH conditions (data not shown).

### Measurement of protein, TAG, and sugar levels

Measurement of protein, TAG, trehalose, glycogen, and glucose was performed as described previously^25^. Haemolymph sample preparation was also performed as previously^25^,^53^. Osmolality was measured using a Micro-Osmometer (Vogel).

### Starvation assay

Starvation assay was performed as described previously^53^,^54^.

### Trehalase activity assay

Two late third instar larvae were rinsed in PBS and homogenized on ice in 100 μl of 10 mM ammonium acetate buffer (pH 5.0) containing 0.1% TritonX-100, 2.5 mM EDTA, and Complete Protease inhibitor (Roche). Five μl of the homogenates was mixed on ice with 10 μl of substrate solution (200 ng/μl trehalose and 20 ng/μl mannitol-1-^13^C (SIGMA) as an internal control in 10 mM ammonium acetate, pH 5.0). The reactions were started at 30 °C and stopped after appropriate times by placing the tubes at 90 °C for 5 min. After cooling to room temperature, assay reactions were mixed with 85 μl of acetonitrile, cleared by centrifugation, and 20 μl of the supernatant was diluted with 20 μl H_2_O. The amounts of trehalose were quantified by LC-MS/MS.

### Quantification of trehalose and glucose by LC-MS/MS

Chromatographic separation was performed on an ACQUITY BEH Amide column (100 mm × 2.1 mm, 1.7 mm particles, Waters) in combination with a VanGuard precolumn (5 mm × 2.1 mm, 1.7 mm particles) using an Acquity UPLC H-Class System (Waters). Elution was performed at 30 °C under isocratic conditions (0.3 mL/min, 70% acetonitrile and 30% 10 mM ammonium bicarbonate, pH 10.0). The mass spectrometric analysis was performed using a Xevo TQD triple quadrupole mass spectrometer (Waters) coupled with an electro-spray ionization source in the negative ion mode. The MRM transitions of *m/z* 341.2 >119 and *m/z* 182.1 >88.9 were used to quantify trehalose and mannitol-^13^C, respectively. Analytical conditions were optimized using standards solution. Sample concentrations were calculated from the standard curve obtained from serial dilution of each standard. The amounts of trehalose were normalized to the levels of mannitol-1-^13^C and further normalized to the levels at time 0 to determine the relative hydrolysis rate in each mutant. TPP activity in trehalose synthesis is Mg^2+^ dependent, whereas the Treh activity is not affected by the addition of EDTA (data not shown). Therefore, we assumed that there was no *de novo* production of trehalose during the incubation period. For the quantification of glucose in the haemolymph, the MRM transition of *m/z* 179.1 >89.0 was used to detect glucose under conditions identical to those described above.

### Measurement of haemolymph volume and desiccation tolerance

Developmental staging and measurement of wet weight were performed as previously described^52^,^53^. The procedures for the measurement of haemolymph volume and the desiccation assay were slightly modified based on previous studies^31^,^55^. For the estimation of haemolymph volume, late third instar larvae were collected, rinsed in PBS, and dried on a Kimwipe. After measuring the wet weight of 8–10 pooled larvae, their cuticle were carefully torn to release the haemolymph on a Parafilm membrane. The released haemolymph from individual larvae was immediately absorbed with a small piece of Kimwipe. The weight of haemolymph was determined by subtracting the weight of the Kimwipe in 1.5 ml tube before and after haemolymph absorption. For measuring dry weight, the wet weight of 8–10 pooled larvae was first measured in 1.5 ml tubes. After being heated at 100 °C for 2 hours, the dry weight of the pooled larvae was measured. Total body water was estimated based on the reduction in mass after dehydration. The proportions of dry weight, total body water, and haemolymph volume were calculated by normalizing them to the values for wet weight.

For the desiccation experiments, early third instar larvae were collected. They were rinsed in PBS and dried carefully on a Kimwipe. The individual larvae were placed in 48 well plates. The plates were covered by a Kimwipe and kept under 10–15% relative humidity in a desiccation chamber containing silica gel for the indicated times. Because of the difficulty in judging the survival rate after desiccation, larvae were rehydrated with a 5% sucrose solution in PBS for 12 hours. The survival rate of larvae was judged based on the movement of the mouth hook under a stereomicroscope.

## Additional Information

**How to cite this article**: Yoshida, M. *et al*. Molecular characterization of *Tps1* and *Treh* genes in *Drosophila* and their role in body water homeostasis. *Sci. Rep*. **6**, 30582; doi: 10.1038/srep30582 (2016).
